# One Health Genomic Perspective on *Pseudescherichia vulneris*: A Neglected Reservoir of Last-Resort Resistance Genes

**DOI:** 10.1007/s00284-026-04948-5

**Published:** 2026-05-19

**Authors:** Anelise S. Ballaben, Julia M. Cabrera, Leandro M. Moreira, Mick Chandler, Alessandro M. Varani

**Affiliations:** 1 Department of Agricultural and Environmental Biotechnology, Faculty of Agricultural and Veterinary Sciences, Sao Paulo State University, Jaboticabal, SP Brazil; 2 Laboratory of Genomics and Bacteria-Environment Interaction, Department of Biological Sciences, Institute of Exact and Biological Sciences, Federal University of Ouro Preto, Ouro Preto, MG Brazil; 3 Department of Biochemistry and Molecular & Cellular Biology, Georgetown University Medical Center, Washington, DC USA

## Abstract

Antimicrobial resistance (AMR) is a critical global threat, often driven by horizontal gene transfer mediated by mobile genetic elements (MGEs) such as plasmids, transposons, and integrons. Among *Enterobacterales*, IncHI2/IncHI2A plasmids are of particular concern, as they combine broad host range, conjugative potential, and mosaic architecture enriched with antimicrobial resistance genes (ARGs), biocide tolerance, and heavy-metal resistance. This study provides the first systematic comparative genomics of *Pseudescherichia vulneris*, an underrecognized yet genomically versatile species at the human–animal–environment interface. All 30 publicly available genomes were analyzed to reconstruct the pangenome, resistome, virulome, and associated MGEs. The pangenome was open, reflecting ongoing diversification and strong potential for horizontal gene acquisition. Resistomes were highly heterogeneous, ranging from minimal repertoires in most animal and environmental isolates to multidrug-resistance profiles in hospital-associated and occasional animal genomes. Clinically significant determinants, including *bla*_KPC-2_*, bla*_KPC-3_*, bla*_CTX-M-9_*,* and *mcr-9*, were frequently linked to MGEs. *bla*_KPC_ alleles were mobilized by Tn*4401*-like elements, while *mcr-9* occurred either within IncHI2/IncHI2A plasmids or integrated into chromosomal contexts, underscoring diverse mobilization routes. In contrast, the virulome was comparatively conserved, dominated by motility, chemotaxis, and siderophore systems, unlike pathogenic *Enterobacterales* that carry broad MGE-associated virulence factors. Co-occurrence analyses showed modular independence between resistance and virulence, with limited overlaps shaped by ecological origins, suggesting that resistome content may adapt to distinctive environments. Collectively, these findings establish *P. vulneris* as a reservoir and conduit of last-resort resistance genes, reinforcing its relevance for One Health surveillance and highlighting the urgent need for its systematic inclusion in global antimicrobial resistance monitoring frameworks.

**Supplementary Information:**

The online version contains supplementary material available at 10.1007/s00284-026-04948-5.

## Introduction

Antimicrobial resistance (AMR) is escalating worldwide and now constitutes a critical threat to public health. Its rise is driven by the widespread use of antibiotics and biocides in clinical and agricultural settings, exacerbated by shortcomings in water, sanitation, and hygiene infrastructure [33]. At the core of this crisis is the horizontal gene transfer (HGT) of antimicrobial-resistance genes (ARGs) mediated by mobile genetic elements (MGEs): plasmids, transposons, insertion sequences, and integrons. Integrons can capture and assemble a wide diversity of ARGs into cassette arrays, but they are not self-mobile. Instead, they are frequently mobilized when embedded within transposons or plasmids, thereby accelerating ARG exchange across ecological boundaries [45]. Recognizing the interconnectedness of humans, animals, and the environment, mediated by HGT and MGEs, underscores the indispensability of the One Health framework for AMR surveillance [23].

For instance, among plasmid incompatibility groups, dual-replicon IncHI2/IncHI2A plasmids are particularly concerning. Their large size, broad host range, abundant cargo of MGEs and efficient conjugal transfer enable them to act as backbones for complex resistance islands carrying ARGs, heavy-metal tolerance loci, and biocide-resistance determinants [3, 14]. Notably, IncHI2/IncHI2A plasmids frequently harbor *mcr-9*, a phosphoethanolamine transferase that can confer inducible resistance to colistin, often a last-resort antibiotic [48].

Unlike *mcr-1*, which is mobilized by the composite transposon Tn*6330* [47], *mcr-9* alleles are embedded in diverse genomic arrangements. They may be bordered by IS*1* or IS*6* family members or captured within class 1 integrons, and their expression often depends on the *qseC*/*qseB* regulatory system [19, 25, 46]. This inducibility complicates phenotypic surveillance, as *mcr-9* carriers can appear colistin-susceptible under standard laboratory conditions. With additional complexity introduced by neighboring MGEs that restructure the genomic context around *mcr-9*, its association with IncHI2/IncHI2A plasmids positions these genes as particularly insidious drivers of antimicrobial resistance dissemination across clinical, veterinary, and environmental settings.

In this broader context, *Pseudescherichia vulneris*, formerly known as *Escherichia vulneris*, emerges as a relevant yet underexplored ARG reservoir. First described in association with opportunistic wound infections and sporadic clinical cases, the species was later reassigned to the genus *Pseudescherichia* following phylogenomic evidence demonstrating substantial divergence from *E. coli* (Alnajar; Gupta, 2017). Although historically regarded as an infrequent opportunistic pathogen, its recognized ecological and clinical distribution has expanded over the past decade.

This species, which exhibits substantial genomic divergence from *E. coli* [4], has been isolated from humans, animals, plants, and contaminated soils, underlining its ecological versatility [29, 52, 55]. Such a broad habitat range, coupled with marked genome plasticity and the absence of systematic surveillance, suggests an enhanced opportunity for *P. vulneris* to acquire, reshuffle, and retain mobile genetic elements across diverse ecological contexts. For instance, a recently described multidrug-resistant *P. vulneris* strain from a healthy domestic cat harbors a dual-replicon IncHI2/IncHI2A plasmid densely packed with ARGs, together with metal-tolerance genes and putative virulence factors, integrated in a mosaic architecture shaped by distinct MGEs [10]. Yet *P. vulneris* remains virtually absent from routine AMR surveillance, a knowledge gap that may obscure its role as a genetic conduit for mobile genetic elements and associated antimicrobial-resistance genes within microbial communities.

More recently, the emergence of a *P. vulneris* strain carrying *bla*_NDM-5_ on an IncX3 plasmid from a veterinary hospital environment (Guangzhou, China) was reported, marking the first detection of a carbapenemase-producing *P. vulneris* in such settings [11]. This finding reinforces the view that the species acts as an environmental bridge for clinically significant ARGs, including carbapenemases, within the One Health continuum.

Therefore, it is possible that *P. vulneris* functions as an environmental intermediary, silently accumulating clinically important ARGs and virulence determinants and subsequently disseminating them to co-occurring *Enterobacterales*. High-risk genes such as *bla*_CTX-M_, *bla*_KPC_, and *mcr*-*9* are likely acquired and maintained within modular platforms, including MGEs and, most prominently, IncHI2/IncHI2A plasmids, reflecting multiple niche-specific events rather than clonal expansion. In this sense, *P. vulneris* may provide a living example of how underexplored taxa can serve as reservoirs and conduits for the mobilome, embodying the genetic promiscuity that underpins emergence of antimicrobial resistance. Adopting a One Health perspective, this study aims to (i) characterize the resistome and virulome of *P. vulneris* across ecological and geographical niches, (ii) delineate the structural organization of associated MGEs, (iii) assess its potential for horizontal transmission, and (iv) clarify its overlooked role within the One Health framework.

## Material and Methods

### Genome Retrieval

A systematic search for *Pseudescherichia vulneris* genomes was conducted in the NCBI Assembly and NCBI Nucleotide databases in January 2025. Searches were performed using the organism’s name “Pseudescherichia vulneris” as the primary query, without restrictions on isolation source or geographical origin. All assemblies taxonomically assigned to *P. vulneris*, including the NCBI reference genome (GCA_902164725.1), were retrieved.

At the time of retrieval, a total of 30 publicly available genome assemblies were identified and included in the study. For a subset of isolates, raw sequencing reads labelled as *P. vulneris* were also available in the Sequence Read Archive (SRA); however, raw reads were not available for all strains. To ensure maximal dataset completeness and comparability, all available assemblies were retained and subjected to uniform quality assessment and downstream analyses. No additional genomes meeting the inclusion criteria were available at the time of the search.

### Read Processing, *de-novo* Assembly and Annotation

For isolates with available raw sequencing data, reads were quality-filtered with fastp v0.20.1 [17] using parameters ‐q 20, ‐l 50, with adapter trimming enabled. Filtered reads were assembled de novo with MEGAHIT v1.2.9 [38] under default parameters. Genome assembly quality was evaluated with CheckM v1.2.2 [43] under the lineage-specific workflow, and contigs < 500 bp or with coverage < 5 × were removed. Both newly assembled genomes and downloaded assemblies were subjected to identical post-processing and quality-filtering criteria to minimize methodological bias. Gene prediction and functional annotation for all genomes, both newly assembled and downloaded, were generated de novo with the NCBI Prokaryotic Genome Annotation Pipeline (PGAP Docker build 2025–03-15), ensuring consistent annotation across the entire dataset.

### Taxonomic Verification

Taxonomic assignments were reassessed using GTDB-Tk under default parameters [16]. Genome classification followed the Genome Taxonomy Database (GTDB) reference framework, which integrates phylogenomic placement and Average Nucleotide Identity (ANI) thresholds for species delineation. ANI values relative to the Pseudescherichia vulneris reference genome were examined to assess species-level coherence.

All genomes were assigned to the genus *Pseudescherichia*. Although a subset displayed ANI values ranging from 91–94% relative to the *P. vulneris* reference genome, indicating genomic heterogeneity within publicly available records, no additional genus-level discrepancies were detected beyond the two excluded datasets. Because the primary aim of this study was comparative genomic characterization rather than formal taxonomic revision, and in the absence of type-strain comparison and phenotypic validation, all retained genomes were analyzed under the original species designation.

### Detection of Antimicrobial-Resistance, Virulence, and Prophages Genes

Protein-coding sequences were screened using ABRicate v1.0.1 (https://github.com/tseemann/abricate) against five antimicrobial resistance (AMR) databases: the “ncbi” database bundled with ABRicate (derived from AMRFinderPlus [27] but not equivalent to the stand-alone AMRFinderPlus implementation; last update: January 2025; ~ 6,000 resistance-associated sequences), CARD v3.2.9 [2] (update: December 2024; ~ 5,000 curated ARGs), ResFinder [9] (last update: November 2024; ~ 3,000 ARG sequences), ARG-ANNOT [31] (last update: 2019; ~ 1,800 ARGs), and MEGARES v3.0 [24] (last update: 2023; ~ 8,000 resistance determinants).

Hits were retained when exhibiting ≥ 80% sequence identity and ≥ 80% coverage. Plasmid replicon types were identified using PlasmidFinder v2.1 [13] (last update: 2024). Virulence-associated genes (VAGs) were detected using ABRicate against the Virulence Factor Database (VFDB) [54] (last update: 2024).

The ABRicate “ncbi” database was selected to ensure methodological consistency across all resistance databases and to allow uniform threshold-based screening. We acknowledge that this database is not identical to the stand-alone AMRFinderPlus software, which applies additional curated rules and hierarchical classification. This choice is further discussed as a methodological limitation.

Presence/absence matrices were constructed for 33 non-redundant ARGs and 29 VAGs detected in at least two genomes.

Prophage regions were identified using PHASTER [6]. Genome assemblies were submitted to the PHASTER web server using default parameters. Regions were classified by PHASTER as intact, questionable, or incomplete based on a composite score that considers similarity to known bacteriophages, gene content, region length, and the proportion of phage-related proteins. Only regions classified as intact were considered for detailed description.

### Mobile Genetic-Element (MGE) Annotation

MGEs, including ISs, Tn, and In, were manually annotated following TnCentral curation guidelines [45] using SnapGene v6.2 (https://www.snapgene.com/) and a TnCentral-based custom library. TnCentral integrates curated ISFinder content and transposon reference sequences, enabling homology-based identification and comparative analysis of transposition modules and structural features. Particular attention was given to the genetic contexts surrounding high-risk loci, containing *bla*_KPC_*, bla*_CTX-M_, and *mcr-9* variants.

### Pangenome Reconstruction and Phylogeny

The pangenome was generated using Panaroo v1.2.9 [51] (–clean-mode strict, core threshold 95%) and analyzed with panstripe v0.3.0 (https://github.com/gtonkinhill/panstripe), both with default parameters. BUSCO analysis was performed on 30 *P. vulneris* genomes, along with *Escherichia coli* str. K-12 substr. MG1655 (NC_000913.3) as the outgroup. BUSCO groups detected in all *P. vulneris* genomes were considered core genes, resulting in 64 single-copy orthologs. The BUSCO_phylogenomics pipeline (https://github.com/jamiemcg/BUSCO_phylogenomics) was used to construct a core-gene supermatrix. A maximum-likelihood phylogenetic tree was inferred from this supermatrix using IQ-TREE v2.1.3 [41] under the GTR + F + I + G4 substitution model, as determined by ModelFinder. Branch support was assessed with 1,000 ultrafast bootstrap replicates, and the tree was rooted using the *E. coli* outgroup. Clade delineation was based on strongly supported nodes (ultrafast bootstrap ≥ 95%) in the maximum-likelihood tree.

### Co-occurrence and Correlation Analysis

Binary presence/absence matrices for the 62 marker genes (33 ARGs, 29 VAGs) across 30 genomes were analyzed in Python 3.9. Absolute co-occurrence counts and Spearman’s ρ were calculated with SciPy v1.10.1. Jaccard distances were converted to similarity scores (1 – distance) using scipy.spatial.distance.jaccard. Gene pairs detected in fewer than five genomes were excluded. Heatmaps were rendered with Matplotlib v3.7.1 and Seaborn v0.11.2, with color intensity indicating correlation magnitude; absolute co-occurrence values were plotted alongside.

All identified VAGs were included without further filtering. In contrast, ARG selection was restricted to genes with established clinical relevance and documented plasmid association, prioritizing determinants involved in resistance to critically important antimicrobial classes and representative of distinct resistance mechanisms within a One Health framework.

### Visualization of Genomic Structures

Circular plasmid maps and linear MGE schematics were generated in SnapGene® v6.2 and Proksee (Grant et al., 2023) then refined in Inkscape v1.2. Heatmaps and presence/absence matrices in the final manuscript were compiled in R v4.2.2 [49] using base functions and ggplot2 for consistent aesthetics.

## Results

### Genomic Landscape, Isolate Distribution and Accessory-Genome Diversity

A search of GenBank (May 2025) retrieved 32 records annotated as *P. vulneris* (18 assembled genomes and 14 raw read sets). After quality control, two datasets (ERR11550397 and SRR18495312), were excluded after taxonomic verification, as they corresponded to *E. coli* and *Franconibacter* spp., respectively. The final genomic catalogue comprised 30 high-quality *P. vulneris* genomes (Table S1). Although all retained genomes were assigned to the genus *Pseudescherichia*, ANI assessment revealed genomic heterogeneity among isolates annotated as *P. vulneris* in public databases, with ten genomes displaying values of 91–94% relative to the reference genome (Table S1). These were retained for comparative analyses under a conservative framework, given the study’s focus on genomic architecture rather than formal taxonomic revision. The 30 analyzed *P. vulneris* genomes span a temporal window from 2006 to 2023 and originate from multiple geographic regions, including North America (n = 11), Europe (n = 7), Asia (n = 6), Africa (n = 3), and Oceania (n = 2), with one isolate lacking precise geographic metadata (Table S1). Most genomes were recovered from clinical or human-associated contexts (50.0%, n = 15), including hospital-related and fecal samples, followed by urban-associated environments (23.3%, n = 7), animal hosts (13.3%, n = 4), and environmental or plant-associated sources (10.0%, n = 3); one genome lacked precise source metadata (3.3%, n = 1). When clinical metadata were available, isolates were linked to opportunistic infection contexts rather than well-defined disease syndromes; however, detailed infection phenotypes were frequently absent or inconsistently reported in public repositories. This heterogeneity and partial absence of metadata reflect common limitations of publicly available genomic datasets and constrain finer epidemiological inference.

Assembly contiguity varied considerably. Four genomes were nearly complete, each comprising two contigs (e.g., GCA_022049045.1, GCA_026651835.1, and GCA_900450975.1) (one chromosome and one plasmid), while one (e.g., CP166292) contained three contigs (one chromosome and two plasmids). The remaining 26 assemblies (~ 87%) were flagged as draft-quality, ranging from moderately fragmented (e.g., GCA_032069025 with 26 contigs) to highly fragmented (e.g., GCA_032096375 with 1,321 contigs). Most assemblies nonetheless exhibited robust quality metrics, with N50 values > 200 kb; for example, GCA_037145055 and GCA_037145235 reached N50 values of 527 kb and 552 kb, respectively.

**Fig. 1:**
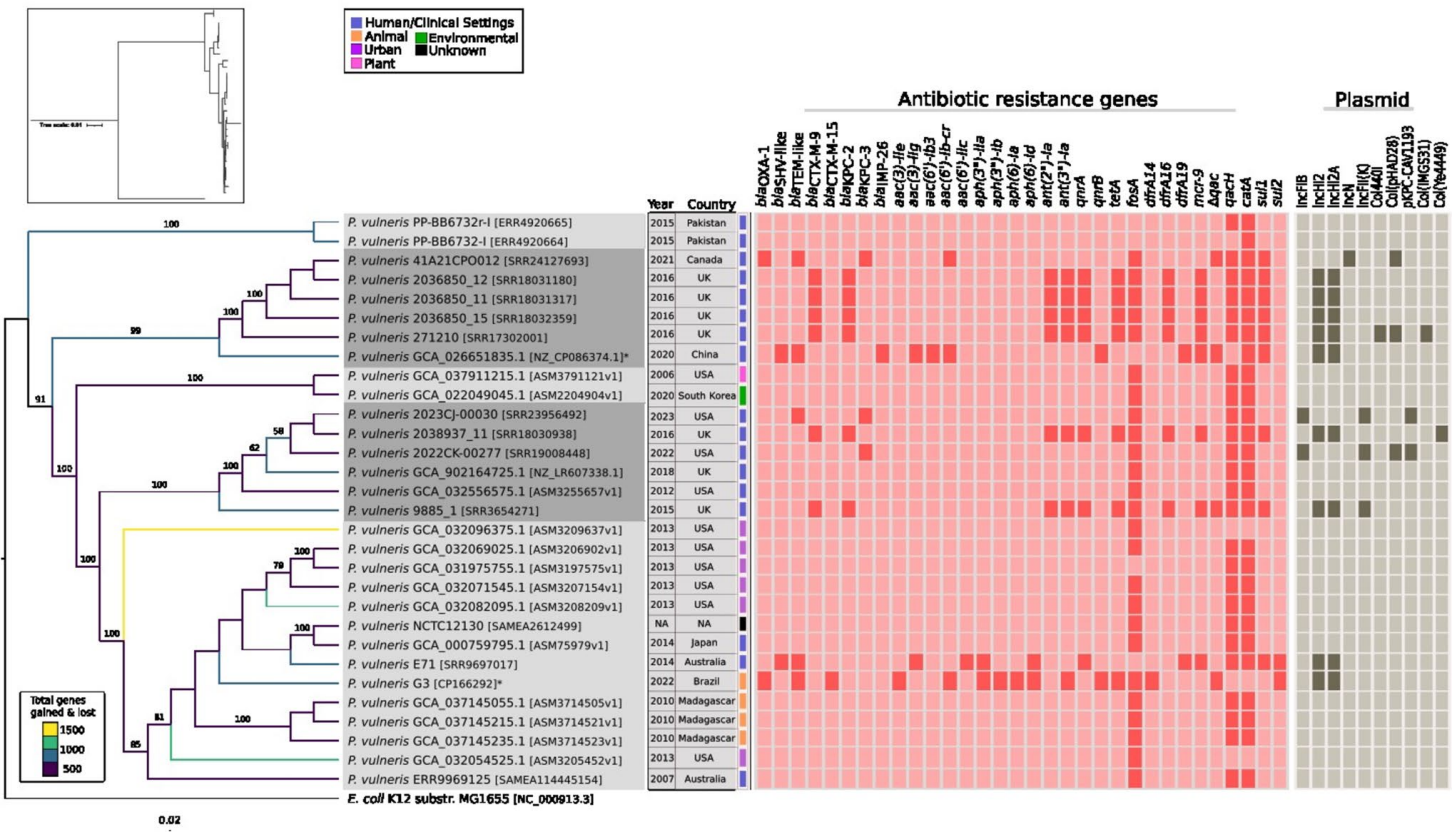
Phylogeny, gene gain–loss dynamics, and distribution of resistance genes and plasmid replicons in Pseudescherichia vulneris. From left to right: a maximum-likelihood phylogeny of 30 *P. vulneris* genomes reconstructed from 64 core genes using IQ-TREE. Branch colors correspond to panstripe analysis, with gradients indicating relative rates of gene gain (yellow) and loss (dark shades); the gain–loss scale is shown in the figure legend. Terminal labels include isolate identifiers with year and country of isolation, and are accompanied by a color strip denoting the ecological source (clinical/human, animal, environmental, urban, plant, or unknown). To the right of the tree, a red–white heatmap depicts the presence (red) or absence of 33 clinically and epidemiologically relevant ARGs. Further to the right, a gray-scale matrix shows the presence of ten plasmid replicon types, with shading indicating detected replicon groups. Together, the visualization highlights phylogenetic structure, ecological origins, and the heterogeneous distribution of ARGs and plasmid backbones across the species

A core-genome alignment of 64 conserved genes was used to construct a maximum-likelihood phylogeny (Fig. 1), which resolved five major clades, each showing distinct gene gain and loss (accessory-genome) profiles. Branch lengths were proportional to substitutions per site, reflecting relative evolutionary divergence among isolates. Although five major clades were resolved with strong bootstrap support, no strict clustering according to geographic origin or ecological source was observed. Some clades showed partial enrichment for hospital-associated isolates or specific regions; however, isolates from different continents and ecological niches were frequently interspersed within the same lineages. This pattern suggests limited phylogeographic structuring and supports the view of *P. vulneris* as a broadly distributed and ecologically versatile lineage. Rarefaction curves did not reach saturation under the current sampling depth, suggesting an open pangenome configuration, although additional genomes will be required to refine this estimate (Figure S1).

Across the dataset, a total of 100 distinct ARGs were detected (Table S2), spanning aminoglycoside, β-lactam, quinolone, macrolide, tetracycline, and sulfonamide classes. In parallel, 29 virulence-associated genes (VAGs) were identified (Table S3), encompassing modules for motility (flagellar and chemotaxis systems), iron acquisition (*fepG*, *entB*), biofilm formation (*csgB*, *csgG*), and immune evasion.

Plasmid replicon typing identified nine incompatibility groups across the 30 genomes, with IncHI2/IncHI2A being the most prevalent, detected in nine hospital-associated isolates. Beyond this group, plasmid diversity in *P. vulneris* was limited: most genomes carried zero to two replicon types, and replicons such as Col(pHAD28), IncFII(K), Col440I, and Col(IMGS31) appeared only sporadically. Notably, IncF-type plasmids, considered as major drivers of ARG dissemination in *Enterobacterales*, were rare, detected in only two genomes, both from hospital-associated isolates.

### Prophage Detection

A total of 30 genomes were screened for prophage regions using PHASTER, and prophage-associated regions were detected in 10 genomes. Among the genomes containing prophage signals, only a subset harbored regions classified as intact according to PHASTER scoring criteria. These intact regions ranged from approximately 19 to 60 kb in length and exhibited high completeness scores (≥ 90), consistent with structurally complete prophages. Intact prophage regions contained a high proportion of phage-related proteins, frequently representing the majority of coding sequences within the region. Gene content included canonical structural and functional phage components such as tail proteins, capsid proteins, terminases, portal proteins, integrases, and lysis-associated proteins, consistent with temperate bacteriophage architecture. The intact regions detected were associated with bacteriophages related to *Enterobacterales*-infecting phages, with BLAST hits distributed across multiple reference phages, reflecting the mosaic nature of prophage genomes. No antimicrobial resistance genes were detected within intact prophage regions. Resistance determinants identified in the dataset were instead predominantly associated with plasmids, integrons, or transposon structures. All intact prophage regions, including genomic coordinates, size, completeness score, and protein counts, are summarized in Supplementary Table S4.

### Diversity, Distribution, and Genomic Context of Clinically Relevant ARGs

A curated panel of 33 clinically and epidemiologically relevant ARGs was screened across the 30 genomes (Fig. 1). The most prevalent were *catA* (n = 28), *fosA* and *qacH* (n = 25 each). Other recurrent loci included *sul1* (n = 9), *mcr-9* (n = 8), *tetA*, *ant(3ʺ)-Ia*, and *qnrA* (n = 7 each), as well as *bla*_CTX-M-9_, *bla*_KPC-2_, *ant(2ʺ)-Ia*, *dfrA16* (n = 6 each), and *bla*_KPC-3_ (n = 3).

While some isolates carried extensive resistomes encompassing ESBLs, carbapenemases, quinolone resistance determinants, and multiple aminoglycoside-modifying enzymes, most animal isolates from Madagascar, as well as several environmental or plant-associated genomes, harbored minimal repertoires, typically limited to *fosA*, *tetA*, and *qacH*. This disparity underscores how resistome size and composition vary sharply according to ecological context.

Such variation is largely driven by MGEs, as inspection of genetic neighborhoods revealed structures consistent with known ARG mobilization pathways. Diverse IS elements flanked carbapenemase, ESBL, together with class 1 integrons assembling modular resistance cassettes in IncHI2/IncHI2A plasmids (e.g., *aac(6’)-Ib3, aac(6’)-Ib-cr5, bla*_OXA-1_*, catB3, qacE* and *sul1* previously shown by [10] and *bla*_IMP-26_ (Figure S2A). These results highlight how *P. vulneris* integrates clinically relevant ARGs within mosaic MGE architectures that may bridge human, animal, and environmental compartments.

**Fig. 2:**
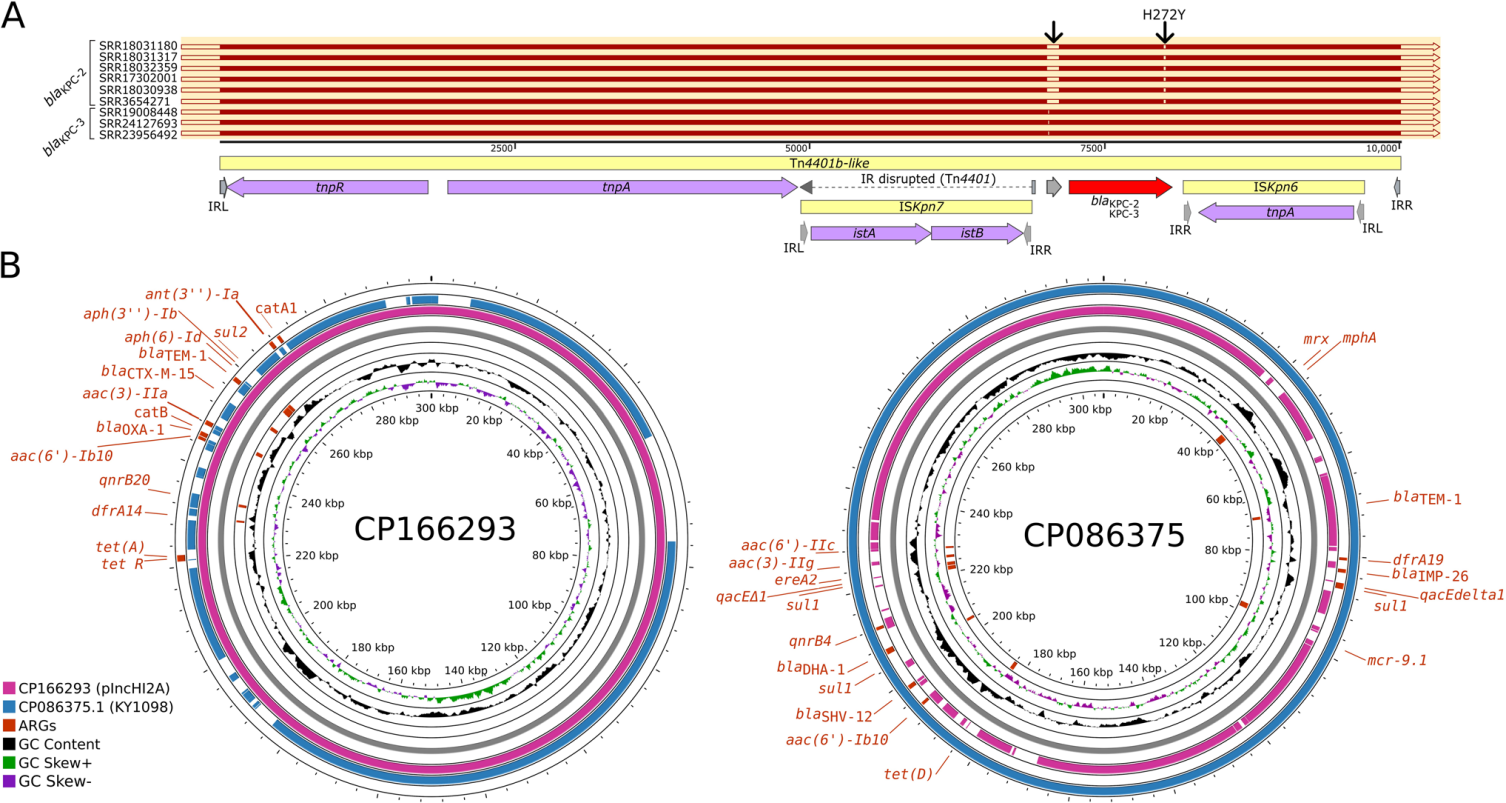
Genetic structures of Tn4401 transposons carrying blaKPC alleles and comparative organization of IncHI2A plasmids in Pseudescherichia vulneris. (A) Comparative analysis of Tn*4401* structures using SnapGene. Six isolates harbored *bla*_KPC-2_, whereas two carried *bla*_KPC-3_. The H272Y substitution, which differentiates the two alleles, is highlighted. Notably, *bla*_KPC-3_–positive isolates contained an exclusive upstream open reading frame encoding a hypothetical protein not observed in *bla*_KPC-2_ contexts. (B) Circular maps of the two fully resolved IncHI2A plasmids identified in *P. vulneris*. Despite sharing a conserved backbone, they displayed marked diversity in accessory resistance regions. The plasmid from China carried mcr-9 together with multiple ARGs, whereas the plasmid from a domestic animal in Brazil lacked *mcr-9*, underscoring independent acquisition events

As an emblematic example, a Tn*4401*-like element was linked to the dissemination of both *bla*_KPC-2_ and *bla*_KPC-3_ (Fig. 2A). This element was identified exclusively in hospital-related isolates, including six from the United Kingdom that carried a hypothetical gene located upstream of *bla*_KPC-2_, and three from North America carrying *bla*_KPC-3_. In the North American isolates, the element occurred within a genomic context compatible with plasmid backbones (apparently not IncHI2/IncHI2A), including *tra* and *vir* genes and proximity to a plasmid *repA*, indicating that the *bla*_KPC_ mobilization may also occur through conjugation (Figure S2B). By contrast, in the United Kingdom isolates it was not possible to resolve the broader genomic context, except for lineage SRR17302001, where the element was clearly embedded within the bacterial chromosome, indicating that Tn*4401*-like transposons can mediate chromosomal integration of carbapenemase genes, providing an additional route for their long-term stabilization beyond plasmid-borne maintenance.

As a contrasting example, six genomes carried *bla*_CTX-M-9_ which is driven by the IS*Ecp1* resident promoter. Unlike other ARGs, this locus was not associated with transposons, integrons, or other recognizable MGEs. Its conserved arrangement across isolates suggests possible chromosomal integration or stabilization within plasmid loci; however, given the fragmented nature of several assemblies, it is not possible to unambiguously determine whether these contexts correspond to chromosomal or plasmid regions, and no clear evidence of recent mobilization was detected.

In the seven genomes carrying *qnrA1*, two main organizational patterns were observed. In five genomes (SRR3654271, SRR17302001, SRR18030938, SRR18031180, and SRR18031317), *qnrA1* was consistently positioned upstream of *ampR*, followed by *hypA* in the reverse orientation and an SMR family transporter in the same orientation as *qnrA1* and *ampR*. This four-gene configuration (*qnrA1–ampR–hypA–SMR*) exhibited highly conserved synteny across the assemblies. By contrast, two genomes (SRR9697017 and SRR18032359) displayed a truncated arrangement in which *qnrA1* was adjacent to *ampR*, but the contigs terminated at the *ampR* locus, precluding resolution of downstream genes (Figure S2C). These cases most likely reflect assembly incompleteness rather than genuine structural variation, given the consistent *qnrA1–ampR* linkage in all seven genomes.

Furthermore, seven genomes carried the tetracycline resistance genes *tetA* and *tetR*. Among them, only the complete genome of *P. vulneris* G3 (CP166292) provided a fully resolved context, where *tetA* and *tetR* were embedded within a degenerated Tn*3*-family element. In contrast, the six draft genomes (SRR3654271, SRR17302001, SRR18030938, SRR18031180, SRR18031317, and SRR18032359) consistently displayed a shorter arrangement consisting of *tetR* and *tetA*, flanked by two reverse orientation IS*6*-family transposases with an intervening small hypothetical ORF (Figure S2D). The recurrent conservation of this IS6–*tetR*–*tetA* module across multiple genomes suggests a common organizational pattern.

#### mcr-9 Plasticity in *Pseudescherichia vulneris*: Dissemination Through IncHI2/IncHI2A Plasmids and Alternative Genomic Contexts

The *mcr-9* gene emerged as the only member of the *mcr* family present in *P. vulneris*. All genomes carrying *mcr-9* are from hospital-related isolates. For instance, *mcr-9.2* was identified in isolates from the United Kingdom (SRR3654271, SRR17302001, SRR18030938, SRR18031180, SRR18031317, SRR18032359), whereas *mcr-9.1* was exclusively detected in plasmid-bearing isolates from China (CP086374.1) and in one Australian strain (SRR9697017). In CP086374.1, the *mcr*-9.1 locus was clearly plasmid-borne within an IncHI2A backbone (Fig. 2B). By contrast, the genomic context of *mcr*-9.1 in SRR9697017 could not be fully resolved due to short-read assembly limitations. Nonetheless, this genome carried a single IncHI2A replicon with the same incompatibility group and plasmid sequence type (ST01) as the Chinese plasmid, suggesting that *mcr*-9.1 is likely embedded in a comparable genetic environment.

The two isolates, CP086374.1 (China) and CP166293 (Brazil), contained fully assembled IncHI2A plasmids, enabling direct comparisons of gene content and organization (Fig. 2B). Both shared a conserved backbone but diverged markedly in accessory regions containing ARG and MGEs: the Brazilian plasmid carried 15 ARGs including those uniquely present in this context (*bla*_CTX-M-15_, *bla*_OXA-1_, *tetA*, *sul2*, *catA1, ant(3″)-Ia, sul2, aph(3″)-Ib, dfrA14*), whereas the Chinese plasmid encoded 16 ARGs, featuring a distinct set of resistance genes (*mcr-9.1*, *bla*_IMP-26_, *bla*_DHA-1_,* aac(6″)-IIc, aac(3)-IIg, ereA2, mphA, mrx, dfrA19, qacEΔ1*, *sul1*, *tetD*). These differences highlight independent acquisition events and reinforce the modular, mosaic nature of IncHI2A plasmids also occurring in *P. vulneris*.

### Co-Occurrence Patterns of ARGs and VAGs

Building on the gene- and plasmid-level analyses, we next explored how ARGs and VAGs are distributed in relation to one another across the 30 genomes. Co-occurrence analysis revealed non-random associations that point to modular resistance–virulence architectures rather than isolated acquisition events.

#### ARG Gene Repertoire and Co-Occurrence

**Fig. 3:**
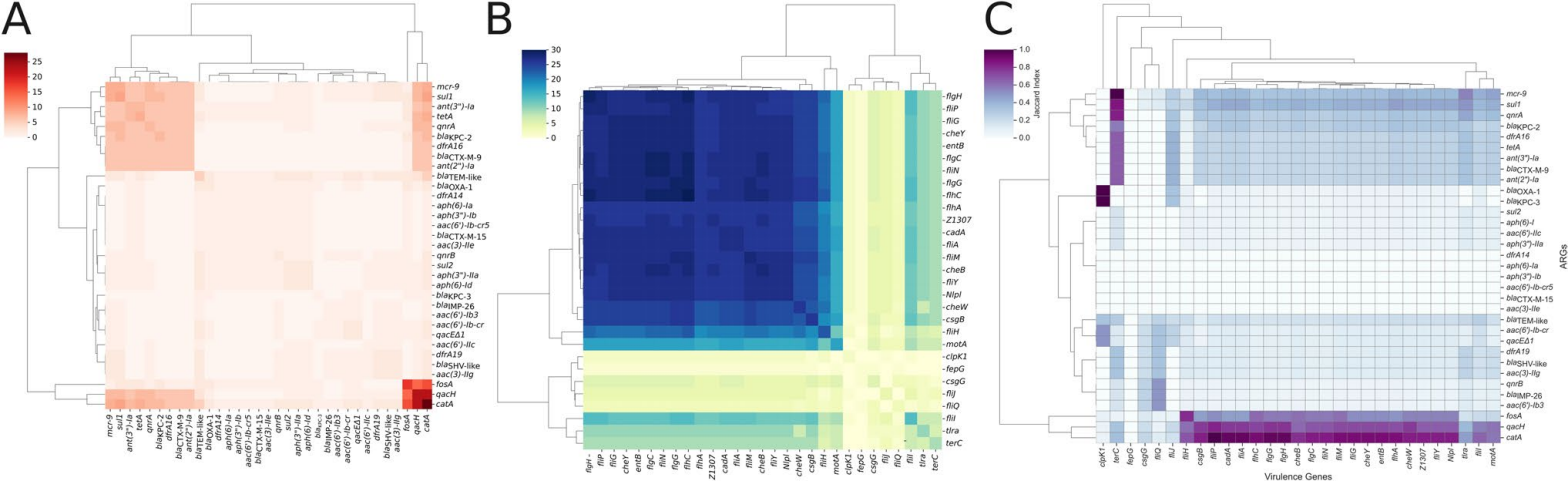
Integrative co-occurrence analyses of ARGs and VAGs in Pseudescherichia vulneris. (A) Pairwise co-occurrence matrix of 33 clinically relevant ARGs across 30 genomes, based on absolute frequency of co-detection. Hierarchical clustering highlights modules of frequently co-occurring ARGs, including a cluster comprising *catA, qacH*, and *fosA*. (B) Co-occurrence matrix of 29 VAGs, showing a conserved module enriched in motility and chemotaxis genes (e.g.,* flhA, fliM, cheY*) and a more variable distribution of accessory virulence-associated factors such as *clpK1* and *tlrA*. (C) Cross-domain co-occurrence matrix between ARGs and VAGs, quantified using Jaccard similarity. Color intensity reflects the degree of co-occurrence between gene pairs. Color scales indicate absolute co-occurrence frequency (A and B) or Jaccard similarity values (C). Hierarchical clustering was applied to both rows and columns. Co-occurrence values represent the frequency with which gene pairs are detected together and do not reflect gene prevalence across genomes

The co-occurrence analysis of the 33 acquired ARGs identified in 30 *P. vulneris* genomes revealed a structured, non-random distribution of resistance determinants (Fig. 3A). Core modules such as *catA*, *qacH*, and *fosA* were repeatedly detected across multiple isolates. High-risk genes (*bla*_CTX-M-9_, *bla*_KPC-2_, and *mcr-9*) were each present in eight genomes, with six isolates (20%; 6/30) carrying all three simultaneously. Additional associations included frequent pairing of *mcr-9* with *sul1* and *ant(3″)-Ia*, linkage of *tetA* with *qnrA*, and co-occurrence of *bla*_KPC-2_ with *bla*_CTX-M-9_. Moreover, *dfrA16* and *ant(2″)-Ia* often clustered alongside β-lactamases and *mcr-9*. Notably, several genomes combined carbapenemases and *mcr-9* with *qac* efflux pumps, pointing to the convergence of antibiotic resistance with tolerance to disinfectants and the potential for cross-selection in clinical, veterinary, and environmental contexts.

The observed co-occurrence patterns do not imply physical co-localization of genes on the same plasmid or replicon across all genomes, particularly given the fragmented nature of most assemblies. Where complete plasmid sequences were available, co-occurring genes were frequently embedded within shared mobile genetic platforms; however, systematic plasmid-level inference was beyond the scope of this study.

#### Virulence Gene Repertoire and Co-Occurrence

In parallel, the distribution of 29 VAGs was evaluated across the dataset, covering structural and regulatory components of the flagellar machinery, chemotaxis, siderophore transport, curli fimbriae, stress response, and metal resistance.

A clear dichotomy between core and accessory components was observed. Genes encoding the flagellar apparatus and chemotaxis system, including *flhC, flgC, flgG, flgH, fliA, fliG, fliM, fliN, cheB, cheW*, and *cheY*, were present in nearly all genomes (≥ 96%), defining a conserved virulence backbone. In contrast, accessory genes linked to environmental persistence and stress adaptation including *clpK1* (heat-shock/stress tolerance), *nlpI* (outer membrane maintenance), *tlrA* and *terC* (tellurite resistance), and *Z1307* (putative virulence factor) showed sporadic distribution across the dataset, with *clpK1* detected in only two genomes. Genes involved in curli fimbriae formation (*csgB* and *csgG*) and siderophore transport (*entB, fepG*) were detected in a substantial subset of genomes but were not universally conserved.

Co-occurrence and hierarchical clustering analysis (Fig. 3B) revealed a prominent module composed of flagellar and chemotaxis genes (*fliA*, *fliG*, *fliH*, *fliM*, *fliN*, *cheB*, *cheW*, *cheY*). A partially overlapping cluster comprised siderophore biosynthesis genes (*entB*, *fepG*) together with curli fimbriae genes (*csgB*, *csgG*). Accessory loci such as *clpK1*, *nlpI*, *tlrA*, *terC*, and *Z1307* showed sporadic co-occurrence and weak connectivity.

#### Integrative ARG–VAG Co-Occurrence

To investigate potential cross-domain associations, we integrated ARG and VAG datasets into a combined co-occurrence analysis (Fig. 3C). Overall, overlap between resistance and virulence modules was limited.

The chloramphenicol resistance gene *catA* displayed strong co-occurrence with a cluster of motility and cell envelope–associated genes (*fliJ–nlpI*, including *fliI* and *motA*). Similarly, *qacH* showed moderate co-association with the same cluster, while *fosA* exhibited weaker associations. The tellurite resistance gene *terC* showed broader connectivity, co-occurring with multiple ARGs including *mcr-9*, *sul1*, *qnrA*, *bla*_KPC-2_, *dfrA16*, *tetA*, *ant(3″)-Ia*, *bla*_CTX-M-9_, and ant*(2″)-Ia*.

By contrast, clinically significant resistance genes such as *bla*_KPC_, *bla*_IMP-26_, and *bla*_CTX-M_ showed little to no overlap with virulence determinants. Their Jaccard index values, generally below 0.3, indicate that these genes are rarely found in the same genomes as virulence factors, suggesting that in *P. vulneris* resistance and virulence evolve largely independently rather than being co-selected within the same mobile platforms.

## Discussion

Despite being long overlooked in antimicrobial resistance (AMR) research, *Pseudescherichia vulneris* displays a genomic configuration characterized by an open pangenome, heterogeneous resistomes, and recurrent association of high-risk ARGs with transmissible plasmids. Analysis of all thirty genomes currently available reveals substantial accessory-genome variability and recurrent linkage of clinically relevant ARGs to IncHI2/IncHI2A plasmids. The additive maximum-likelihood phylogeny further resolved five well-supported clades with branch lengths proportional to evolutionary divergence; however, no strict clustering according to geographic origin or ecological source was observed. Although partial enrichment of hospital-associated isolates occurred within certain clades, genomes from distinct continents and ecological niches were frequently interspersed. This limited phylogeographic structuring suggests that lineage diversification in *P. vulneris* is not strongly constrained by regional or ecological boundaries, a pattern consistent with reports of other opportunistic *Enterobacterales* in which gene flow and plasmid exchange transcend geographic compartments [15, 18, 28, 53]. These broad-host-range plasmids frequently carry multiple ARG classes together with disinfectant, biocide, and heavy-metal tolerance loci, and are widely distributed among *Enterobacterales* worldwide [4]. Although this is not the first report of IncHI2 or IncHI2A plasmids in *P. vulneris* [10], our findings demonstrate that they were detected in multiple independent genomes within the current dataset in this lineage, positioning the species as an additional reservoir of resistance genes. Given their conjugative potential [26], these plasmids provide a robust platform for the long-term maintenance and horizontal transmission of last-resort resistance determinants across distinct *Enterobacterales* species from different ecological compartments [3].

Beyond plasmid carriage, *P. vulneris* harbors diverse ARG repertoires shaped by integron cassettes and transposons directly associated with gene mobilization. For instance, *bla*_KPC-2_ and *bla*_KPC-3_ were embedded in Tn*4401*-like elements, which are recognized for mobilizing *bla*_KPC_ genes at high frequency [20], whereas *bla*_IMP-26_, *qacEΔ1*, and *sul1* were carried within class 1 integrons. Similarly, *qnrA1* was consistently linked to *ampR*, and in most genomes also to *hypA* and a SMR family transporter, forming a conserved arrangement previously reported in *Enterobacterales* and often associated with IS*CR1* and class 1 integrons [30, 34]. In addition, *tet*(A) and *tetR* were consistently associated with flanking IS*6*-family transposases. Although the surrounding IS*26* copies were arranged in inverted orientation rather than the direct configuration typical of IS*26*-mediated pseudo-compound transposons, similar IS*26*-flanked resistance regions in *Enterobacterales* are known to facilitate gene capture, recombination, and stabilization [8, 10]. By contrast, *bla*_CTX-M-9_ showed conserved synteny with no recognizable MGEs detected in the surrounding contigs; however, plasmid localization cannot be excluded due to assembly fragmentation. Together, these observations reflect two contrasting modes of resistance maintenance in *P. vulneris*: dynamic mobilization via transposons and integrons versus apparent long-term stabilization.

Interestingly, several hospital-associated genomes carried high-burden resistance profiles that included *bla*_CTX-M-9_ and *mcr-9*, whereas most animal and environmental isolates encoded only baseline determinants such as *fosA*, *tetA*, and *qacH*. This contrast suggests that *P. vulneris* adapts its resistome according to ecological context, accumulating clinically significant ARGs in hospital environments while maintaining minimal repertoires in non-clinical settings. The importance of One Health genomic surveillance has been widely emphasized for well-characterized human pathogens and zoonotic agents, including *Clostridioides difficile*, Influenza A viruses, *Acinetobacter baumannii*, and *E. coli* [15, 18, 28, 53]. In this context, such ecological plasticity reinforces the need to consider *P. vulneris* within a One Health surveillance framework, as its ability to shift resistome content across environments positions it as a silent but relevant intermediary in AMR dissemination.

Notably, the triad *bla*_KPC-2/3_, *bla*_CTX-M-9_, and *mcr-9* occurred together in six genomes (20%), highlighting convergence of resistance to carbapenems, ESBLs, and colistin within single isolates. This co-occurrence mirrors plasmid-borne dissemination of ESBLs and carbapenemases in *Klebsiella pneumoniae* [5]. Moreover, the geographic distribution of *mcr-9* in this dataset aligns with global surveys, with *mcr-9.1* predominating in Asia and the Americas and *mcr-9.2* more common in Europe [48]. The recurrent association of *mcr-9* with IncHI2/IncHI2A plasmids underscores its dissemination potential across diverse *Enterobacterales* lineages, while occasional chromosomal integration highlights multiple routes of mobilization and maintenance. From a clinical perspective, the detection of *bla*_KPC_, *bla*_CTX-M-9_, and *mcr-9* within the same genomes is concerning, as it may compromise the efficacy of carbapenems, extended-spectrum cephalosporins, and colistin, agents frequently reserved for multidrug-resistant *Enterobacterales* infections. Although phenotypic data were unavailable, the genomic presence of these determinants suggests potential therapeutic limitations and reinforces the need for accurate species-level identification and susceptibility testing in clinical laboratories.

Compared to classical *Enterobacterales*, the resistome of *P. vulneris* appears comparatively simpler and seems to rely predominantly on plasmid acquisition. While *K. pneumoniae* and *E. coli* typically combine multiple resistance layers, including chromosomal mutations (*gyrA, parC*), plasmid-borne ESBLs (*bla*_CTX-M_), diverse carbapenemases (KPC, NDM, OXA-48-like), and multiple *mcr* alleles [35, 37], and *Enterobacter* spp. possess inducible chromosomal AmpC β-lactamases supplemented by plasmid-borne determinants [21, 50], *P. vulneris* shows no intrinsic β-lactamase arsenal. Instead, it appears to rely on acquisition of IncHI2/IncHI2A plasmids carrying *bla*_KPC-2/3_ and *mcr-9* as its principal route to clinically relevant resistance. This plasmid-dependent and comparatively “lighter” resistome mirrors its opportunistic ecology and suggests that its epidemiological impact becomes significant only when high-risk plasmids converge within the same host genome.

The integrative co-occurrence analysis further highlighted modular independence between resistance and virulence. Core virulence modules, including motility and chemotaxis, were consistently conserved, while siderophore transport and curli fimbriae formed a secondary cluster. Clinically critical resistance determinants (*bla*_KPC_*, bla*_IMP-26_*, bla*_CTX-M_*, mcr-9*) rarely overlapped with virulence loci, in contrast to *E. coli* ExPEC lineages where IncF plasmids often co-carry resistance and adhesins such as *fimH* [12, 36]. Only limited bridges were observed, notably *catA* and *qacH* with motility or envelope clusters, and *terC* spanning multiple ARGs. This pattern mirrors modularity reported in environmental *Enterobacterales* [40], suggesting that resistance and virulence in *P. vulneris* evolve under distinct selective pressures. Prophage regions were also detected in a subset of genomes, highlighting an additional layer of genome plasticity. However, no antimicrobial resistance genes were identified within intact prophage regions, suggesting that, in contrast to plasmids and transposons, bacteriophage-mediated transfer does not appear to represent a major route for ARG dissemination in the current dataset.

In addition, *P. vulneris* virulence content contrasts with that of major pathogens. *E. coli* (ExPEC) typically harbors dense pathogenic islands encoding adhesins (*fimH, papG*), toxins (*hlyA, cnf1*), siderophores (aerobactin, salmochelin), and secretion systems (T3SS, T6SS) [7, 22]. *K. pneumoniae* often acquires hypervirulent plasmids with regulators (*rmpA/rmpA2*), potent siderophores (*iuc, iro, ybt*), and genotoxins such as *clb* [32, 44]. *Enterobacter* spp., particularly the *E. cloacae* complex, maintain robust repertoires enriched in adhesion, biofilm, and immune evasion [40]. By contrast, *P. vulneris* displays a fragmented and ecologically oriented virulome, largely restricted to motility, chemotaxis, environmental sensing, and basic adhesion. The absence of pathogenic islands or horizontally acquired high-virulence modules suggests an environmentally adapted, opportunistic lifestyle.

In line with our observations, Cai et al. [11] recently described the emergence of *bla*_NDM-5_-carrying *P. vulneris* and *Pantoea dispersa* isolated from a veterinary hospital environment in China, expanding the known diversity of carbapenemase contexts in this species [11]. Their detection of an IncX3 plasmid carrying *bla*_NDM-5_ further supports the conclusion that *P. vulneris* can acquire and maintain distinct plasmid backbones for disseminating high-risk ARGs. Together with our findings, this data confirms that *P. vulneris* is not a passive environmental commensal but an adaptable vector linking clinical, veterinary, and environmental reservoirs within the One Health network.

Recent studies have demonstrated that the genomic epidemiology of neglected or understudied bacterial species can be advanced rapidly through the systematic integration of publicly available genomes, even when datasets are limited in size or completeness. From a One Health perspective, such approaches have proven effective in revealing overlooked reservoirs and transmission platforms that bridge clinical, veterinary, and environmental compartments. A recent example illustrates how comparative genomics can rapidly illuminate the epidemiology of an understudied pathogen by leveraging fragmented yet globally distributed genomic data [1].

By systematically analyzing all genomes currently available for Pseudescherichia vulneris, this study establishes a much-needed foundation for understanding its mobilome and resistance potential. Our findings are consistent with broader surveys showing that ARG–virulence co-localization is more common in pathogens associated with human and animal hosts than in environmental species [42], and that horizontal transfer is shaped by both genetic compatibility and ecological connectivity [39]. Paradoxically, such gaps explain why *P. vulneris* has long been overlooked in AMR research. Despite this neglect, our analyses uncovered high-risk ARGs, stabilized *mcr-9* loci, and IncHI2/IncHI2A plasmids with mosaic architectures, underscoring that *P. vulneris* is unlikely to be only a passive commensal.

### Limitations

This study is subject to several limitations inherent to analyses based exclusively on publicly available genomic data. First, the number of *P. vulneris* genomes currently available remains limited (n = 30), and the dataset is unevenly distributed across ecological contexts and geographic regions. Such sampling bias may influence estimates of pangenome openness, ARG prevalence, and phylogeographic structure.

Second, most genomes analyzed were draft assemblies (26/30), which restrict precise delineation of MGE boundaries and limit definitive assignment of ARGs to chromosomal or plasmid locations. Although replicon typing and contextual analysis were used to infer plasmid associations, complete plasmid reconstruction and statistical ARG–plasmid linkage analyses were not feasible for the majority of isolates due to assembly fragmentation.

Third, prophage detection was performed using *in-silico* prediction tools and limited to intact regions as defined by PHASTER scoring criteria. While no ARGs were identified within intact prophage regions, the fragmented nature of several assemblies may reduce sensitivity for detecting incomplete or mosaic phage-associated mobilization events.

Fourth, metadata associated with publicly deposited genomes were frequently incomplete or inconsistently reported, limiting fine-scale epidemiological interpretation, including infection type, transmission pathways, and clinical outcomes. In addition, phenotypic antimicrobial susceptibility data were unavailable for most isolates, precluding direct genotype–phenotype correlation, particularly for inducible resistance determinants such as *mcr-9*.

Finally, clade delineation was based on strongly supported phylogenetic nodes rather than formal population-structure clustering algorithms. While sufficient for comparative genomic inference at the current sampling depth, higher-resolution population analyses will require expanded datasets and standardized lineage-typing schemes.

Despite these limitations, the consistency of mobilome-associated resistance patterns observed across independent genomes supports the robustness of the main conclusions and highlights the need for expanded genomic surveillance of this neglected species.

## Conclusions

*Pseudescherichia vulneris* emerges as an underrecognized yet genomically versatile member of *Enterobacterales*, equipped with a broad mobilome and capable of harboring clinically significant resistance determinants. Its pangenome remains open, reflecting ongoing diversification, while the resistome spans from baseline determinants (*fosA, tetA, qacH*) in non-clinical isolates to multidrug-resistance profiles in hospital-associated and occasional animal strains. Importantly, *bla*_KPC-2/3_, *bla*_CTX-M-9_, and *mcr-9* were recurrently detected, with *mcr-9* embedded in IncHI2/IncHI2A plasmids and, in some cases, chromosomal contexts, underscoring multiple routes of mobilization and stabilization.

In contrast to epidemic pathogens such as *Escherichia coli* and *Klebsiella pneumoniae*, *P. vulneris* lacks an intrinsic β-lactamase arsenal or high-virulence pathogenicity islands. Its resistome appears comparatively “lighter” and plasmid-dependent, suggesting that its epidemiological relevance becomes critical only when high-risk plasmids converge within the same genome. This opportunistic profile reflects an environmentally adapted lifestyle but also highlights its capacity to act as a silent reservoir for last-resort resistance genes.

From a One Health perspective, the recurrent association of high-risk ARGs with transmissible IncHI2/IncHI2A plasmids, structures enriched in disinfectant, biocide, and heavy-metal tolerance loci, represents a durable chassis for cross-sector dissemination. Recognizing *P. vulneris* in genomic surveillance is therefore essential, as expanding monitoring to include this neglected lineage will improve the anticipation of multidrug-resistant platforms bridging clinical, veterinary, and environmental compartments.

## Supplementary Information

Below is the link to the electronic supplementary material.

Supplementary file 1.

Supplementary file 2.

Supplementary file 3.

Supplementary file 4.

Supplementary file 5.

Supplementary file 6.

## Data Availability

No datasets were generated or analysed during the current study.
