# Supplementary figures and images for: One Health Genomic Perspective on Pseudescherichia vulneris: A Neglected Reservoir of Last-Resort Resistance Genes
Source: Curr Microbiol. 2026 May 19;83(7):370. doi: 10.1007/s00284-026-04948-5 (PMC13186809; doi:10.1007/s00284-026-04948-5)

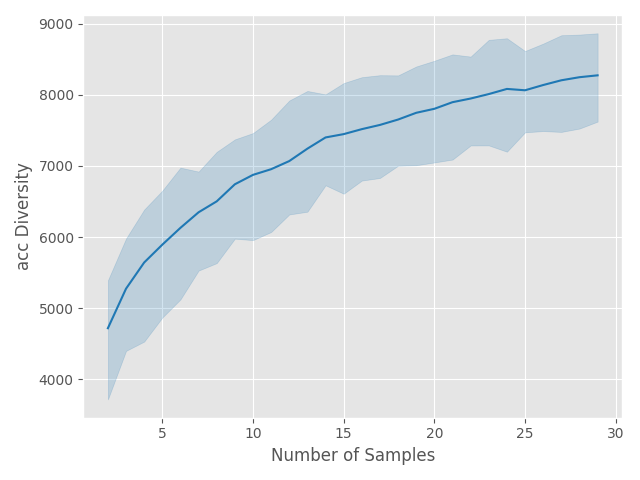

Supplement: Supplementary file 1 [file 284_2026_4948_MOESM1_ESM.png]

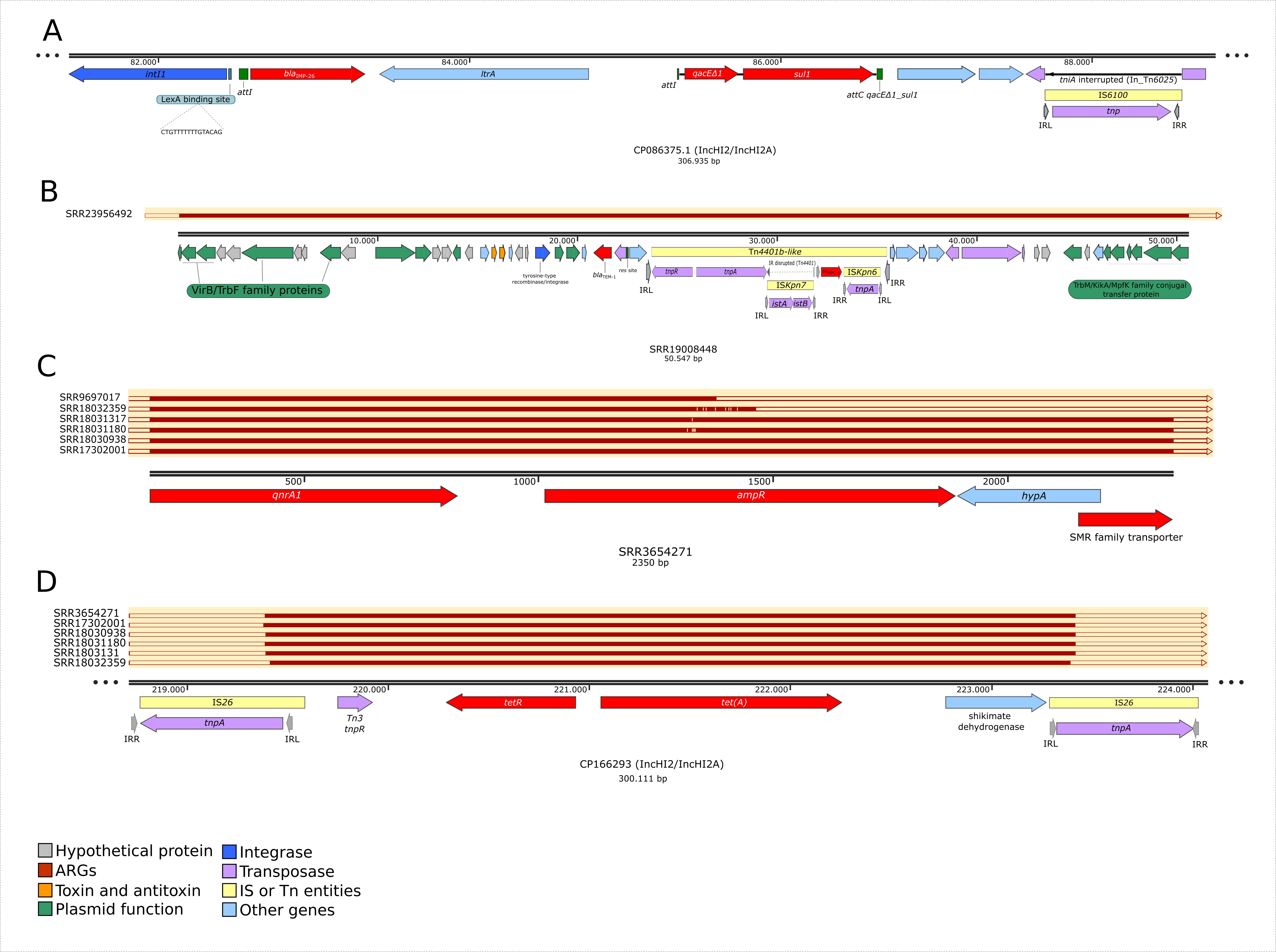

Supplement: Supplementary file 2 [file 284_2026_4948_MOESM2_ESM.jpg]
